# Supplementary material for: eIF4A1 Is a Prognostic Marker and Actionable Target in Human Hepatocellular Carcinoma
Source: Int J Mol Sci. 2023 Jan 20;24(3):2055. doi: 10.3390/ijms24032055 (PMC9917075; doi:10.3390/ijms24032055)
Supplement: Supplementary file 1 [file ijms-24-02055-s001.zip › ijms-2121872-supplementary.pdf]

# SUPPLEMENTARY DATA

The analysis used data from 47 patients with human hepatocarcinoma (35 males and 12 females). The variables have been analyzed using the Statistical Package for Social Science (SPSS, version 22.0, Chicago, IL, USA).

## 1. Descriptive statistics

| Sex | Number (%) | Mean survival (SD) |
|---|---|---|
| Male | 35 (74.5) | 35.76 (25.67) |
| Female | 12 (25.5) | 32.89 (26.72) |
| Total | 47 | 35.03 (25.67) |

Mean survival (all patients) is 35.03 months (SD 25.67)

| Sex | eIF4A1 (SD) | eIF4A2 (SD) | DDX3 (SD) |
|---|---|---|---|
| Male | 0.624 (0.495) | 0.571 (0.406) | 0.666 (0.699) |
| Female | 0.660 (0.615) | 0.541 (0.301) | 0.330 (0.246) |
| Total | 0.633 (0.522) | 0.564 (0.379) | 0.580 (0.630) |

## 2. eIF4A1 in hepatocarcinoma

Low and high eIF4A1 protein levels were recoded into binary variables (0/1) using the median value (eIF4A1 = 0.523) as the cut-off. The whole dataset was then divided into 23 patients with eIF4A1 below the median, and 24 patients above the median. Statistical comparison between the two groups was performed using the **log-rank test**:

| Marker | Number of patients (%) | Mean survival in months (SD) | Log-rank test |
|---|---|---|---|
| eIF4A1 < 0.523 | 23 (48.9) | 45.68 (25.76) | – |
| eIF4A1 ≥ 0.523 | 24 (51.1) | 24.82 (21.48) | **0.005** |
| Total | 47 | 35.03 (25.67) | |

Conclusion 1: **Patients with eIF4A1 above the median 0.523 survive on average shorter than patients with eiF4A1 below 0.523.**

## 3. eiF4A2 in hepatocarcinoma

Low and high eIF4A2 protein levels were recoded into binary variables (0/1) using the median value (eIF4A2 = 0.492) as the cut-off. The whole dataset was then divided into 23 patients with eIF4A2 below the median, and 24 patients above the median. Statistical comparison between the two groups was performed using the **log-rank test**:

| Marker | Number of patients (%) | Mean survival in months (SD) | Log-rank test |
|---|---|---|---|
| eIF4A2 < 0.492 | 23 (48.9) | 34.34 (23.78) | – |
| eIF4A2 ≥ 0.492 | 24 (51.1) | 35.69 (27.86) | 0.741 |
| Total | 47 | 35.03 (25.67) | |

Conclusion 2: **Patients with eIF4A2 mRNA above the median 0.492 do not survive on average shorter than patients with eIF4A2 below 0.492.**

## 4. DDX3 in hepatocarcinoma

Low and high DDX3 protein levels were recoded into binary variables (0/1) using the median value (DDX3 = 0.433) as the cut-off. The whole dataset was then divided into 23 patients with DDX3 below the median, and 24 patients above the median. Statistical comparison between the two groups was performed using the **log-rank test**:

| Marker | Number of patients (%) | Mean survival in months (SD) | Log-rank test |
|---|---|---|---|
| DDX3 < 0.433 | 23 (48.9) | 27.99 (26.74) | – |
| DDX3 ≥ 0.433 | 24 (51.1) | 41.77 (23.19) | 0.142 |
| Total | 47 | 35.03 (25.67) | |

Conclusion 3: **Patients with DDX3 above the median 0.433 survive on average longer than patients with DDX3 below 0.433 but without statistical significance.**

## 5. Survival analysis (univariate)

| Variable | Survival (months) (SD) | p-value |
|---|---|---|
| *Age (years)* | | |
| < 65 | 34.54 (24.90) | 0.900 |
| ≥ 65 | 35.50 (26.92) | |
| *Sex* | | |
| Female | 35.76 (25.67) | 0.742 |
| Male | 32.89 (26.72) | |
| *Cirrhosis* | | |
| No | 34.06 (18.19) | 0.881 |
| Yes | 35.36 (28.00) | |
| *Etiology* | | |
| HBV | 31.54 (25.34) | 0.245[#] |
| HCV | 32.33 (19.51) | |
| Ethanol | 35.58 (31.40) | |
| *Size* | | |
| < 3 cm | 43.07 (26.38) | 0.212 |
| ≥ 3 cm | 32.27 (25.22) | |
| *Serum AFP* | | |
| < 300 ng/ml | 42.12 (30.19) | 0.156 |
| ≥ 300 ng/ml | 31.01 (22.29) | |
| *Tumor grade* | | |
| II | 42.23 (26.14) | 0.137[#] |
| III | 38.17 (29.32) | |
| IV | 23.85 (15.61) | |
| *eIF4A1* | | |
| *< 0.523* | 45.68 (25.76) | **0.004** |
| *≥ 0.523* | 24.82 (21.48) | |
| *eIF4A2* | | |
| *< 0.492* | 34.34 (23.78) | 0.860 |
| *≥ 0.492* | 35.69 (27.86) | |
| *DDX3* | | |
| *< 0.433* | 27.99 (26.74) | 0.065 |

| ≥ *0.433* | 41.77 (23.19) | |
|---|---|---|

#One-way ANOVA

In the univariate analysis, a significant difference in survival was found only for the eIF4A1 protein.

## 6. Multivariate Cox regression analysis

A multivariate Cox proportional hazard model was constructed with survival as the outcome variable. The eiF4A1, eiF4A2, and DDX3 variables were included in three different models, together with all covariates (full model). Hazard ratios (HRs) and their 95% confidence intervals were calculated, and the Wald test was used for model testing.

| **Covariates** | **Full model (HR and 95% CI)** | **Full model (HR and 95% CI)** | **Full model (HR and 95% CI)** |
|---|---|---|---|
| Age | 1.016 (0.973–1.061) | 1.025 (0.982–1.070) | 1.019 (0.974–1.067) |
| Male sex | 1.111 (0.450–2.744) | 1.263 (0.537–2.970) | 1.417 (0.613–3.275) |
| Cirrhosis (y/n) | 1.229 (0.504–2.995) | 0.770 (0.322–1.846) | 0.856 (0.350–2.095) |
| *Etiology* | | | |
| HBV | Reference | Reference | Reference |
| HCV | 1.077 (0.366–3.166) | 0.594 (0.214–1.652) | 0.733 (0.255–2.102) |
| Ethanol | 1.289 (0.430–3.864) | 0.889 (0.318–2.492) | 1.551 (0.521–4.617) |
| Diameter > 3 cm | 1.852 (0.804–4.265) | 1.749 (0.758–4.038) | 1.475 (0.672–3.240) |
| AFP > 300 ng/ml | 3.107 (1.277–7.564) * | 2.019 (0.841–4.848) | 2.387 (0.977–5.834) |
| Grade | | | |
| II | Reference | Reference | Reference |
| III | 0.500 (0.199–1.258) | 0.983 (0.438–2.206) | 1.017 (0.450–2.298) |
| IV | 1.749 (0.712–4.295) | 2.454 (1.048–5.751) * | 2.790 (1.166–6.677) * |
| eIF4A1 ≥ 0.523 (median value) | **4.751** (1.944–11.61) ** | – | – |
| eIF4A2 ≥ 0.492 (median value) | – | 1.110 (0.578–2.133) | – |
| DDX3 ≥ 0.433 (median value) | – | – | **0.420** (0.201–0.876) * |

*p<0.05; **p<0.0001

Conclusion: **Levels of eIF4A1 protein greater than 0.523 are significant predictors of mortality in patients with hepatocellular carcinoma. Levels of DDX3 protein greater than 0.433 significantly predict more prolonged survival in patients with hepatocellular carcinoma.**

**Supplementary Table S1.** Clinicopathological features of HCC patients.

| Variables | Features | |
|---|---|---|
| | HCCB | HCCP |
| Number of patients | | |
| Male | 17 | 18 |
| Female | 5 | 7 |
| Age (Mean ± SD) | 64.77 ± 8.7 | 66.32 ± 10.6 |
| Etiology | | |
| HBV | 9 | 9 |
| HCV | 7 | 11 |
| Ethanol | 3 | 3 |
| N/A | 3 | 2 |
| Cirrhosis | | |
| + | 16 | 19 |
| - | 6 | 6 |
| Tumor size | | |
| > 5 cm | 15 | 19 |
| < 5 cm | 7 | 6 |
| Edmondson and Steiner grade | | |
| I | 0 | 0 |
| II | 7 | 6 |
| III | 11 | 9 |
| IV | 4 | 10 |
| Serum alpha-fetoprotein level (ng/ml) | | |
| > 300 | 13 | 17 |
| < 300 | 9 | 8 |
| Survival after partial liver resection (months) | | |
| Means ± SD | 57.3 ± 18.5 | 15.4 ± 9.6 |

**Abbreviations:** N/A: Not applicable, SD: Standard deviation

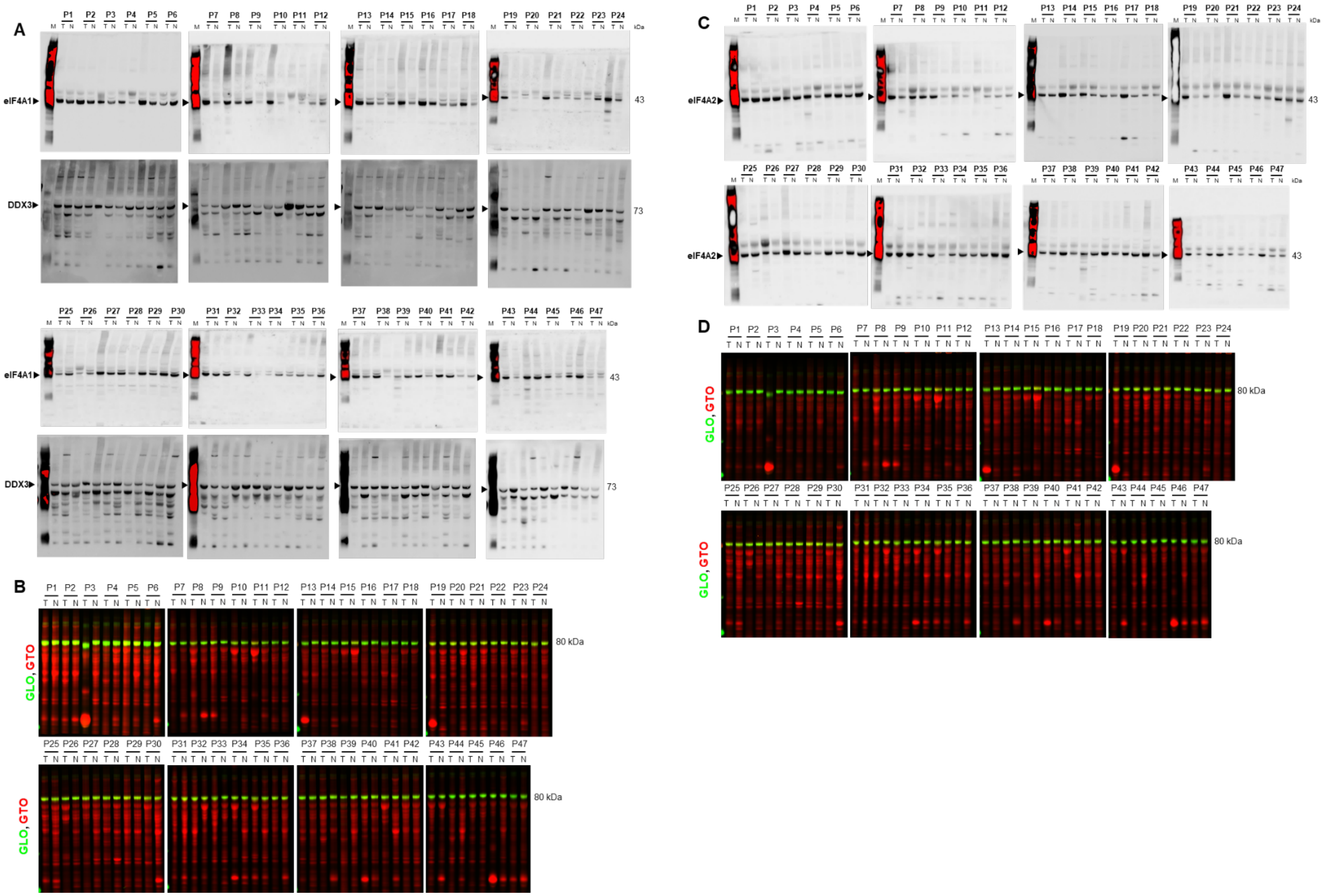


**Supplementary Figure S1:** Details of Western Blot data (uncropped membanes) in human HCC samples and paired non-tumor tissues. (**a**) Chemiluminescent images of eIF4A1 and DDX3 Western Blots. (**b**) Fluorescence images of eIF4A1 and DDX3 PAA gels with GLO (green) and GTO (red). (**c**) Chemiluminescent images of eIF4A2 Western Blots. (**d**) Fluorescence images of the eIF4A2 PAA gel with GLO (green) and GTO (red). N: Human normal tissue; T: Human HCC tumor tissue; P: Patient; GLO: Gel loading control at 80 kDa (green); GTO: Total protein signals (red).

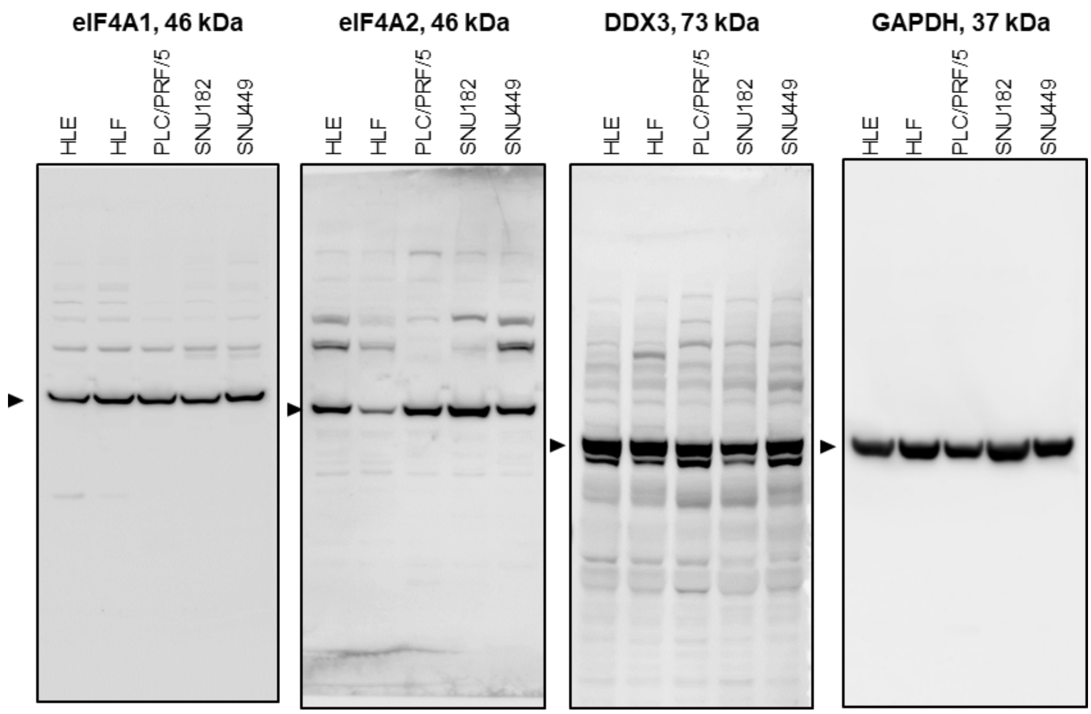


**Supplementary Figure S2:** Detailed Western Blot images (uncropped membranes) of eIF4A1, eIF4A2, DDX3, and GAPDH expression in different cell lines.

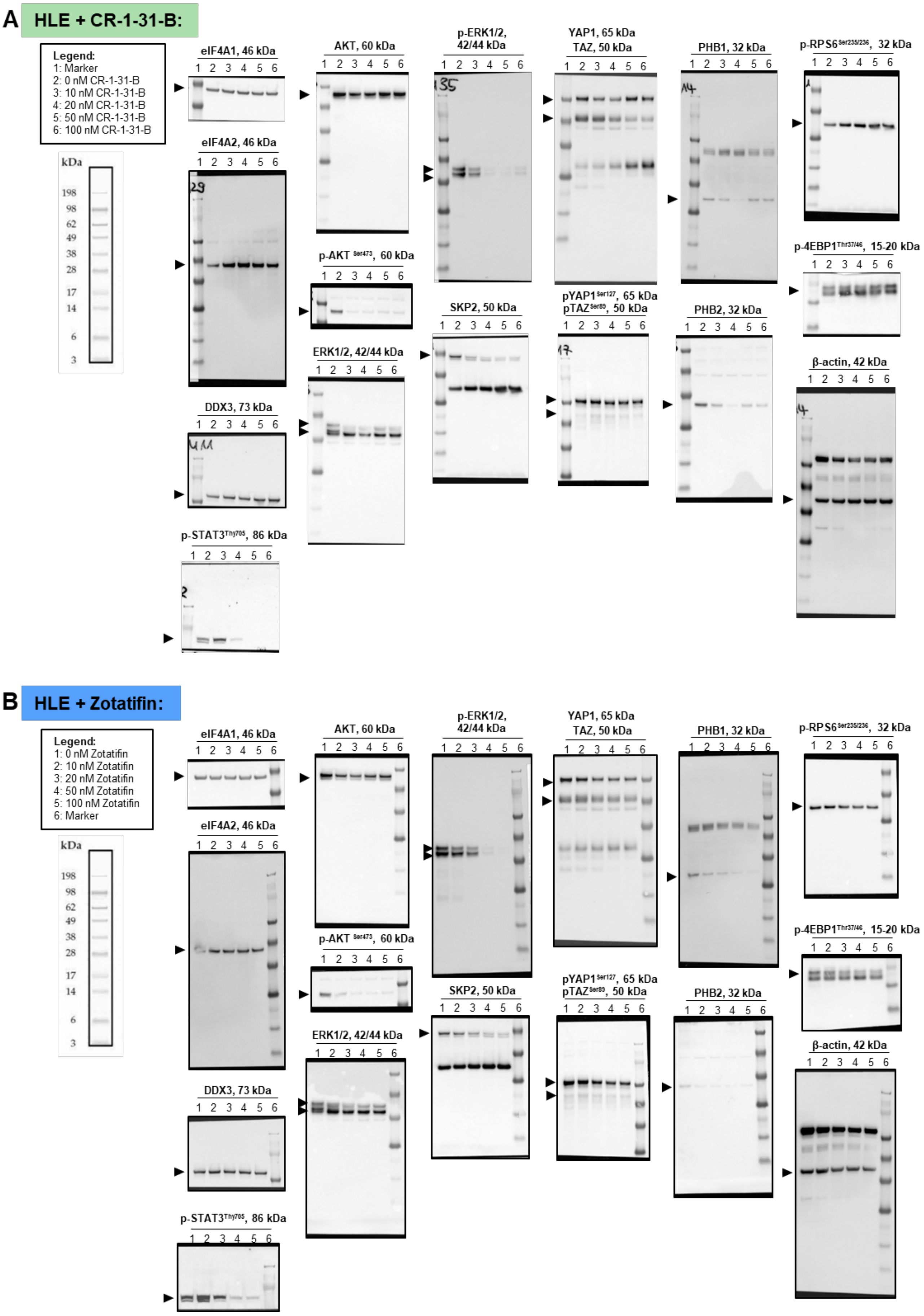


**Supplementary Figure S3:** Details of Western Blot data (uncropped membranes) in HLE cells.

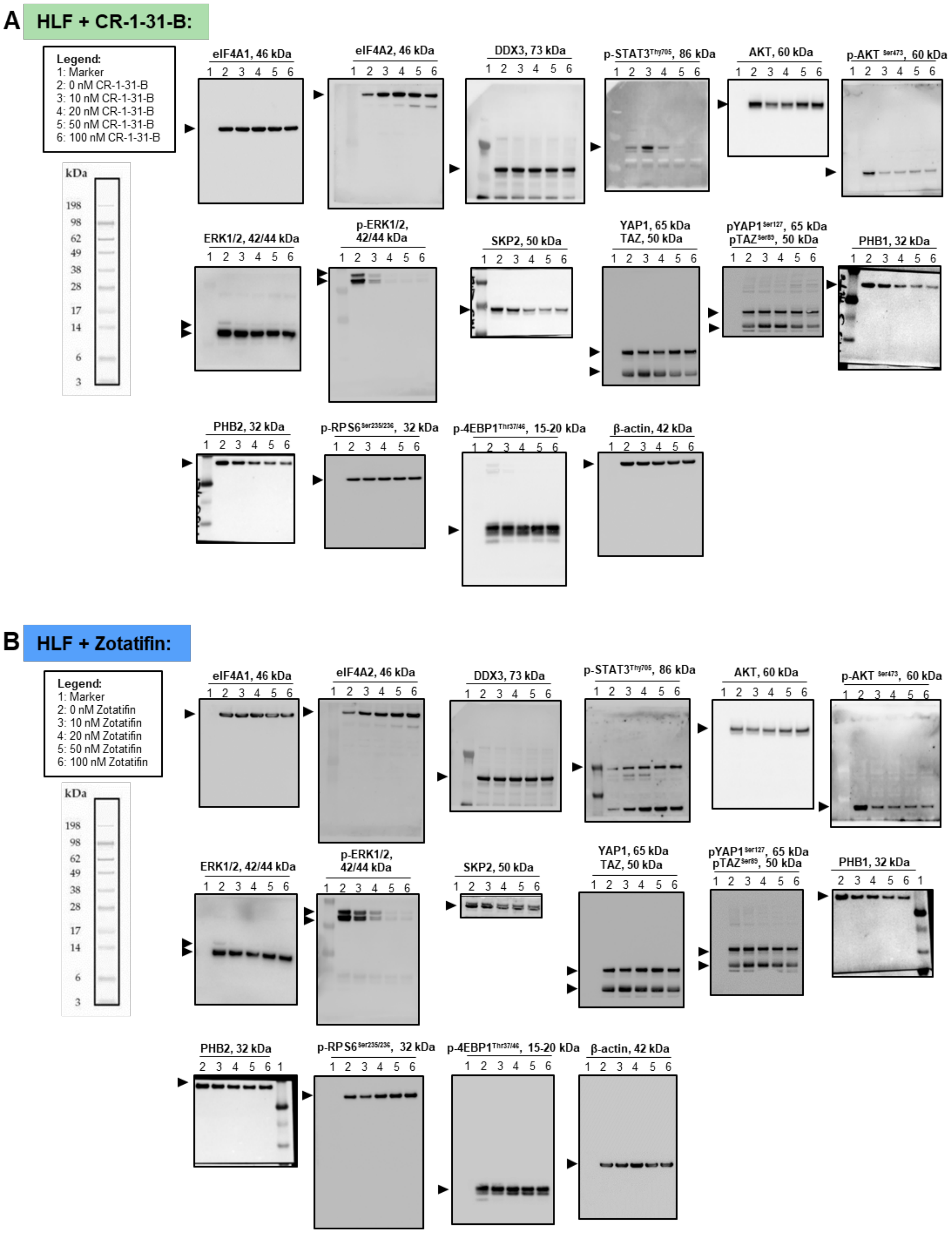


**Supplementary Figure S4:** Details of Western Blot data (uncropped membranes) in HLF cells.

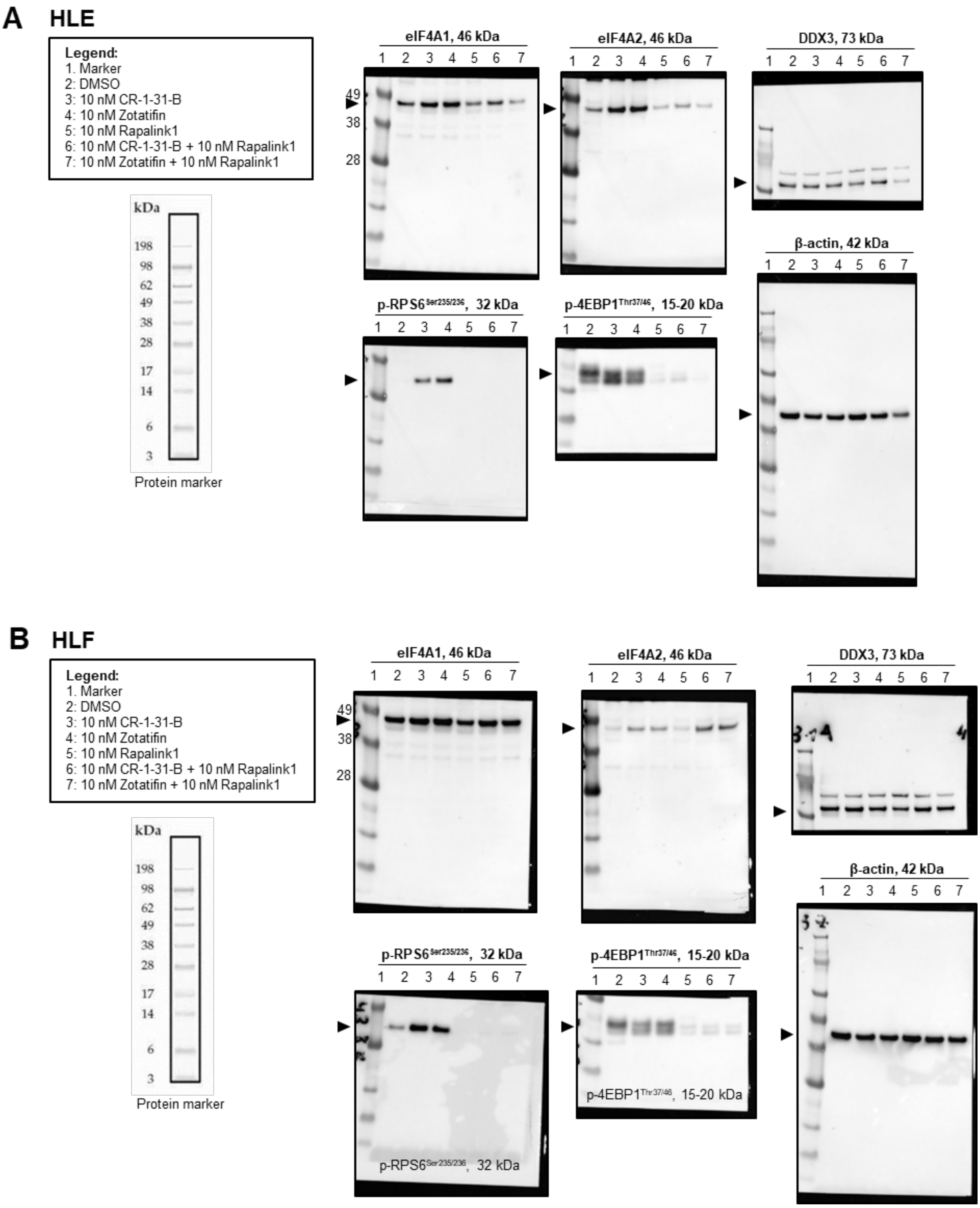


**Supplementary Figure S5:** Details of Western Blot data (uncropped membranes) in HLE and HLF cells.
